# Central role of PD-L1 in cardioprotection resulting from P2Y_4_ nucleotide receptor loss

**DOI:** 10.3389/fimmu.2022.1006934

**Published:** 2022-09-28

**Authors:** Michael Horckmans, Esteban Diaz Villamil, Mariaelvy Bianchini, Lucas De Roeck, Didier Communi

**Affiliations:** ^1^ Institute of Interdisciplinary Research, Institut de Recherche Interdisciplinaire en Biologie Humaine et Moléculaire (IRIBHM), Free University of Brussels, Brussels, Belgium; ^2^ Institute for Cardio-vascular Prevention, Ludwig-Maximilians-Universität (LMU), Munich, German

**Keywords:** cardioprotection, cardiac adipose tissue, PD-L1, P2Y receptor, adiponectin, ischemia

## Abstract

A better understanding of the immune function of pericardial adipose tissue is essential to adapt treatments after myocardial infarction. We showed previously that inactivation of mouse P2Y_4_ nucleotide receptor induces adiponectin overexpression and protection against myocardial infarction. We investigated here the inflammatory state of pericardial adipose tissue in ischemic P2Y_4_-deficient mice. We demonstrated that P2Y_4_-deficient mice displayed adipocyte beiging with increased PD-L1 expression and a higher number of regulatory leukocytes in their pericardial adipose tissue after left anterior descending artery ligation, compared to wild type mice. Effectively, a higher level of anti-inflammatory M2c macrophages and regulatory T cells was observed in pericardial adipose tissue of P2Y_4_ KO mice and correlated with reduced post-ischemic expansion of fat-associated lymphoid clusters. Interestingly, the anti-inflammatory effects observed in P2Y_4_ KO mice, were no more observed in P2Y_4_/adiponectin double KO ischemic mice. Finally, the reduction of T cell infiltration and cardiac fibrosis observed in P2Y_4_-deficient heart was lost after injection of anti-PD-L1 blocking antibody in ischemic mice. The present study defines P2Y_4_ as a regulator of PD-L1 and adiponectin, and as a potential target for anti-inflammatory therapies to improve myocardial infarction outcome. The combined effect of P2Y_4_ loss on adipocyte beiging and regulatory leukocyte increase highlights this nucleotide receptor as an important player in post-ischemic cardiac response.

## Introduction

Recent advances in the management of acute myocardial infarction have led to improved cardiac outcomes. However, patients with obesity continue to experience a higher risk of adverse events after myocardial infarction (MI), including recurrent ischemia compared with lean patients, or even death. Despite the adverse outcomes associated with increased adipose tissue volume, patients are not a homogeneous group, with important differences in comorbidities, glucose metabolism, insulin resistance, and other clinical factors. Such differences can markedly alter the risks of individual patients and their potential benefit from various treatment strategies. Highlighting these diverse patient- or treatment-specific adverse outcomes could improve the identification of patients at higher risk and the follow-up after MI. Contrariwise, lower risk patients may prefer and benefit from a more conservative treatment. We have shown previously that aggressive anti-inflammatory strategies reducing neutrophil influx in order to limit acute post-ischemic tissue injury might also inhibit the subsequent healing process (1), illustrating that the cardiac healing response is tightly regulated.

Recently, pericardial adipose tissue (PAT) was identified as an immunologically active organ in which lymphocyte subsets contribute to rapid immune responses with the coordination of immune cell activation within fat-associated lymphoid clusters (FALCs) (2). FALCs are found in almost every adipose tissue, with the highest density observed in PAT (2). FALCs are in direct contact with the adipocytes, and composed by leukocytes, mainly B and T cells (3). MI induces an acute increase of inflammatory cytokines in the circulation and an expansion of lymphocytes in the FALCs. We have shown previously that FALCs and PAT communicate to other tissues as bone marrow to modulate granulopoiesis, and outcome after MI (4). PAT volume is highly associated with the severity of coronary artery disease (5, 6) and can contribute to affect long‐term mortality of patients with systolic heart failure (7). Although recent studies have focused on the possible links between PAT and cardiometabolic diseases, the factors involved are not well identified.

Highly vascularized and innervated, white adipose tissue (WAT) plays a key homeostatic role, not only by warranting energy storage but also as a paracrine and endocrine organ that releases several active substances, such as adiponectin. It has been well established that the consumption of a high-fat diet has many direct effects on adipose tissues. WAT undergoes various cellular and structural remodeling processes, including vascularization and recruitment of inflammatory cells. Appropriate plasticity seems to protect against metabolic and cardiovascular disorders. However, when obesity-associated inflammatory state is sustained, the adaptive homeostatic mechanisms fail, leading to WAT dysfunction, characterized by impaired secretion of adipokines, such as adiponectin. There is evidence that dysfunctional WAT can be balanced by the activation of UCP1^+^ brown adipocytes that can be found among WAT (8). Increasing the number of these adipocytes, called beige adipocytes, has been suggested as a potential therapeutic approach to treat human obesity/diabetes (8–11).

We identified previously a role of nucleotide P2Y_4_ receptor in cardioprotection (12) and PAT formation (13). P2Y_4_ subtype is a UTP receptor in human, originally cloned in our laboratory (14), and equally activated by ATP and UTP in mouse (15). Weshowed that mouse P2Y_4_ inactivation induces protection against myocardial infarction and adiponectin overexpression (12, 13). We decided to investigate here the potential regulation of adipocyte beiging and regulatory leukocyte populations in PAT of P2Y_4_-deficient ischemic mice.

## Materials and methods

### Ischemia *in vivo* experiments: LAD ligation

Adiponectin knockout (KO) mice named B6;129-Adipo^tm1Chan^/J were purchased at JAX, The Jackson Laboratory (Bar Harbor, ME, USA). C57BL/6J P2Y_4_ KO and P2Y_4_/adiponectin double KO mice were generated in our laboratory. We used randomly male and female mice, aged from 11 to 13 weeks. MI was induced by permanent ligation of the left anterior descending coronary artery, as previously described (14, 15). Mice were anesthetized with midazolam (5 mg/kg), medetomidine hydrochloride (0.5 mg/kg) and fentanyl (0.05 mg/kg), intubated, and ventilated with a MiniVent mouse ventilator (Harvard Apparatus, Holliston, MA, USA). Left thoracotomy was performed in the fourth left intercostal space, and the pericardium was carefully incised to maintain the integrity of the PAT. The chest wall and skin were closed with 5‐0 silk sutures (Covidien, Dublin, Ireland). After surgery, naloxone hydrochloride (1.2 mg/kg), flumazenil (0.5 mg/kg) and atipamezole hydrochloride (2.5 mg/kg) were injected to reverse the effect of anesthesia. Postoperative analgesia (buprenorphine, 0.1 mg/kg) was given for the first 12 hours after surgery. There was no significant difference in the low mortality rate for the different types of mice used in the present study, as well as for both male and female mice. The survival rate in ischemic animals was over 90%, 7 days after LAD ligation.

### RNA-sequencing experiments

RNA-sequencing experiments were performed on a pool of RNAs extracted from the PAT of 3 WT and a pool of RNAs extracted from the PAT of 3 P2Y_4_ KO ischemic mice (24h post-MI). RNAs were isolated from freshly harvested PAT using the RNeasy Mini Kit (Qiagen, Hilden, Germany) after cell lysis in TRIzol Reagent Solution (Invitrogen, Thermo Fisher Scientific, Waltham, MA, USA). 1μg/50μL of RNA was engaged and the quality was checked using a Bioanalyzer 2100 (Agilent Technologies, Santa Clara, CA, USA). cDNA libraries were obtained using the TruSeq Stranded mRNA Library Prep kit (NuGEN Technologies, San Carlos, CA, USA) following manufacturer recommendations. The multiplex libraries (18pM) were loaded on flow cells and sequences were produced using a HiSeq PE Cluster Kit v4 and TruSeq SBS Kit v3‐HS from a HiSeq 1500 (Illumina, San Diego, CA, USA). Approximately 25 million paired‐end reads per sample were mapped against the mouse reference genome (GRCm38.p4/mm10) using STAR software to generate read alignments for each sample. Annotations Mus_musculus GRC38.87.gtf were obtained from ftp.Ensembl.org. After transcripts assembling, gene level counts were obtained using HTSeq. Genes with CPM >0.5 and a fold change P2Y_4_ KO/WT ≥ 2 or ≤ 0.5 were considered. Gene Ontology enrichment analysis was performed with DAVID software. Enriched biological processes were selected by the software to have a significant (p<0.05) modified Fisher Exact P‐Value, or EASE score.

### Quantitative RT‐PCR experiments

Total mRNAs were extracted through homogenization of PAT of P2Y_4_ KO and WT mice in a glass‐teflon tissue grinder in TRIzol reagent followed by purification with RNeasy kit column. mRNA was reverse transcribed using random hexamers and Superscript II Reverse Transcriptase (Invitrogen, Thermo Fisher Scientific, Carlsbad, CA, USA). RT‐PCR amplification mixtures contained 10 ng template cDNA and primers specific for Ucp1, Cited1, Prdm16, Il33, and Ccl24 synthetized from their corresponding gene sequences. Reactions were run on a 7500 Fast Real Time PCR System (Applied Biosystems, Foster City, CA, USA). qPCR data, expressed as Ct, were normalized for each gene to Rpl32 housekeeping gene to obtain relative expression values.

### Histological experiments on cardiac adipose tissue

PAT from control and ischemic mice were harvested after PFA 4% perfusion. Paraffin cross-sections (5 µm) were cut. Dewaxed sections were incubated with rabbit anti‐UCP1 (Abcam, Cambridge, UK), according to the manufacturer’s protocol. Endogenous peroxidase was blocked with 3% hydrogen peroxide in methanol and incubated with normal rabbit serum (1:75) for 20 min to reduce nonspecific background. The tissue sections were incubated with primary antibodies against UCP1 overnight at 4°C. After incubation with secondary antibody (IgG biotin conjugated (1:200; Vector Laboratories, Newark, CA, USA), enzymatic reaction was performed to reveal peroxidase with Sigma Fast 3,3′‐diaminobenzidine (Sigma‐Aldrich, St. Louis, MO, USA) as substrate. Finally, sections were counterstained with hematoxylin and mounted.

For fibrosis quantification, paraffin cross-sections (8 µm) of infarcted hearts were cut, fixed in Bouin solution (Sigma-Aldrich) and stained with Masson’s trichrome (Sigma-Aldrich), according to the manufacturer’s protocol. Fibrosis area was quantified as the relative area of blue staining (collagen) compared to the left ventricle surface, as an average of three sections per heart at different levels, using ImageJ software.

### Immunofluorescence experiments

Whole-mounted mouse PAT was fixed in 2% PFA, stained overnight with antibodies against Programmed death ligand 1 (PD-L1) (BioLegend, USA) and perilipin (Abcam, UK), then optically cleared with RapiClear 1.47 (SUNJin Lab, South Korea) for 6 hours. Samples were acquired as tile-scan overviews with a Leica Thunder Imager (Leica Microsystems, Germany). For higher optical resolution imaging, 3D image stacks were acquired using a Leica SP8 3X confocal microscope (Leica Microsystems, Germany) equipped with a tunable white light laser. Image 3D reconstructions were generated with Imaris 8 (Oxford Instruments, UK).

Frozen sections of mouse hearts were stained with antibody against CD3 (BD Biosciences, Franklin Lakes, NJ, USA). T cell density was quantified using ImageJ software by examining 10 fields per section at 100x magnification, in a blinded fashion. Sections were counterstained with Hoechst to visualize the entire population of cell nuclei within each myocardial section. For all histological examinations, at least 3 sections per mouse were analyzed.

### Isolation and differentiation of cardiac adipose‐derived stem cells

cADSCs were isolated from the stromal vascular fraction of PAT, as previously described (13). PAT was minced and incubated in collagenase A solution (2.5 g/L collagenase A, 50 μg/ml DNase; Roche, Basel, Switzerland) at 37°C for 45 min. The suspension was fractionated into mature adipocytes and stromal vascular fraction by centrifugation at 500 g for 5 min. ADSCs were cultured for 3 days in proliferation medium (DMEM (Gibco, Thermo Fisher Scientific, Bleiswijk, The Netherlands) containing 3% newborn calf serum and 1% penicillin/streptomycin) and for 7 days in adipogenic differentiation medium (proliferation medium supplemented with 50 μM indomethacin, 1 μM dexamethasone, 0.5 mM isobutylmethylxanthine, and 5 μg/ml insulin). The presence of lipid droplets was revealed by Oil Red O staining (Sigma‐Aldrich, St Louis, MO, USA) and their size was quantified and reported in 4 categories (0-2 µm, 2-4 µm, 4-6 µm and > 6 µm) using ImageJ software.

### Flow cytometry experiments

PAT of control mice or mice subjected to LAD were collected after the sacrifice of the mice and perfusion with PBS to remove peripheral cells. PAT was then finely minced and digested in collagenase A solution (2.5 g/L collagenase A and 50 μg/ml DNase (Roche, Basel, Switzerland)) at 37°C for 45 min. The single cell suspension was rinsed, resuspended in PBS supplemented with 3% FBS and CD16/CD32 Fc‐block (clone 2.4G2; BD Pharmingen, BD Biosciences, Franklin Lakes, NJ, USA) and stained with a mix of fluorochrome‐conjugated antibodies for 45 min on ice. Antibodies used were CD45 (clone 30F11), CD11b (clone M1/70), F4/80 (clone BM8), CD3 (clone 17A2), as well as CD25, Foxp3, CD44, CD62L, CD4, CD8, annexin V for lymphocyte population analysis (all from BioLegend) and CD206 (BioLegend) and MertK (AF591, R&D, Abingdon, UK) for macrophage population analysis. Flow cytometry experiments were also performed using differentiated cADSCs with PD-L1 antibody (BioLegend). Data were acquired on a Fortessa (BD Biosciences, Franklin Lakes, NJ, USA), and analysis was performed with FlowJo software (Ashland, OR, USA).

### Use of anti-PD-L1 blocking antibody

To evaluate the importance of PD-L1 effects in our model, anti-PD-L1 blocking antibody (B7-H1, clone 10F.9G2) (BioXCell, Lebanon, NH, USA) and its control IgG2b,κ antibody were used at different steps of the study. For quantification of T cell in PAT and ischemic hearts, 7 days post-MI, anti-PD-L1 blocking antibody or control antibody (10 µg/g) were injected 1h before LAD ligation and then at days 1, 3 and 5 post-LAD ligation.

### Statistics

All the data obtained are expressed as mean ± SEM, and statistical analysis was performed with GraphPad Prism software (version 6; GraphPad Software, San Diego, CA, USA). Endpoint comparisons between 2 groups were performed using unpaired 2-tailed Student’s *t* test. For multiple comparisons in parallel repeated-measures studies, ANOVA was used with Bonferroni *post-hoc evaluation*. A 2-tailed p < 0.05 was considered as significant.

## Results

### Loss of mouse P2Y_4_ induces beiging of pericardial adipose tissue

**Figure 1:**
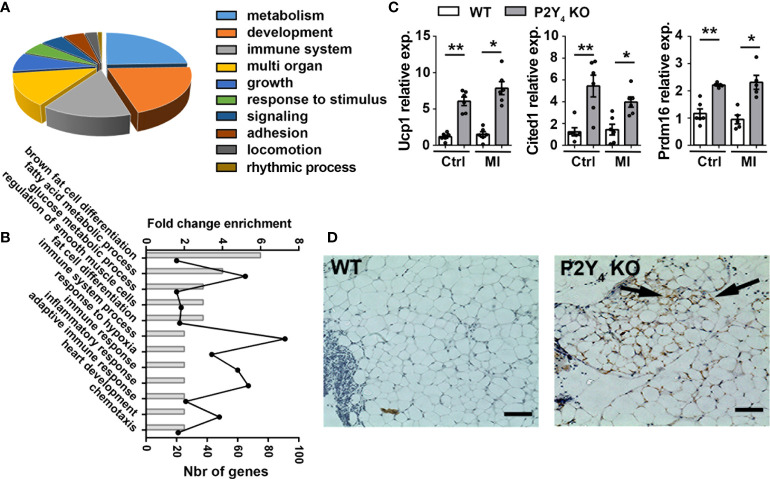
Loss of mouse P2Y_4_ induces beiging of pericardial adipose tissue. **(A)** Representation of the biological processes related to the genes differentially expressed between PAT of P2Y_4_ KO and WT ischemic mice (24h post-MI) in RNA-sequencing experiments. **(B)** Selection of biological processes revealed as enriched for a differentially expressed number of genes (Nbr of genes) between PAT RNA of P2Y_4_ KO and WT ischemic mice, after a Gene Ontology enrichment analyses performed with DAVID software. **(C)** qPCR quantification of Ucp1, Cited1 and Prdm16 mRNAs in PAT of P2Y_4_ KO and WT control (Ctrl) mice or 24h after LAD ligation (myocardial infarction (MI)), normalized to Rpl32 housekeeping gene (n = 6). **(D)** UCP1 staining performed by immunohistochemistry on paraffin‐embedded sections of PAT of WT and P2Y_4_ KO ischemic mice (24h post-MI) and counterstained with hematoxylin/eosin (scale bar = 50 μm). Examples of regions positive for UCP1 staining are indicated by black arrows and images were taken at original magnification 100x. Data represent mean ± SEM. *p < 0.05; **p < 0.01.

To identify target genes in PAT potentially involved in cardioprotection, we performed RNA-sequencing experiments using RNA samples pooled from PAT of 3 P2Y_4_ KO and 3 WT ischemic mice, 24h after left anterior descending coronary artery (LAD) ligation. The RNA-sequencing datasets and a complete list of the genes differentially expressed is now available with GEO accession number GSE211768. We observed that 2268 genes and 1433 genes were respectively, up-regulated or down-regulated in the PAT of P2Y_4_ KO mice with a ratio ≥ 2 or ≤ 0.5, compared to the PAT of WT mice (data not shown). A gene enrichment analysis with DAVID software on the RNA-sequencing data obtained from P2Y_4_ KO versus WT PAT revealed that several biological processes linked to metabolic process, development, immune response and inflammation were enriched (**Figures 1A, B**). **Table 1** includes the differentially expressed genes related to the biological process group “brown fat cell differentiation” displayed in (**Figure 1B**). Among the genes regulated in P2Y_4_ KO PAT, we decided to focus on up-regulated genes linked to adipocyte beiging, named Ucp1, Cited1 and Prdm16. We analyzed the expression of these beige adipocyte markers in the PAT of P2Y_4_ KO and WT mice by qPCR. We confirmed higher levels of Ucp1, Cited1 and Prdm16 expression suggesting adipocyte beiging in P2Y_4_ KO compared to WT PAT, both in basal and ischemic conditions (**Figure 1C**), and regions showing UCP1 expression were then identified by immunostaining in PAT sections from ischemic P2Y_4_ KO mice (**Figure 1D**).

**Table 1:** Genes involved in brown adipocyte differentiation and differentially regulated in the PAT of P2Y_4_ KO compared to the PAT of WT mice.

Gene symbol	Gene name	RatioP2Y_4_ KO/WT
Ucp1	uncoupling protein 1 (mitochondrial proton carrier)	168,12
Mb	myoglobin	41,59
Dio2	deiodinase iodothyronine type II	12,33
Adrb3	adrenergic receptor beta 3	7,37
Slc2a4	solute carrier family 2 (facilitated glucose transporter) member 4	5,61
Cited1	Cbp/p300-interacting transactivator with Glu/Asp-rich carboxy-terminal domain 1	3,50
Scd1	stearoyl-Coenzyme A desaturase 1	3,24
Adig	adipogenin	3,17
Pparg	peroxisome proliferator activated receptor gamma	2,84
Fabp3	fatty acid binding protein 3 muscle and heart	2,74
Adipoq	adiponectin C1Q and collagen domain containing	2,69
Gata2	GATA binding protein 2	2,67
Prdm16	PR domain containing 16	2,41
Ebf2	early B cell factor 2	2,26
Nudt7	nudix (nucleoside diphosphate linked moiety X)-type motif 7	2,19
Aldh6a1	aldehyde dehydrogenase family 6 subfamily A1	2,18

List of genes identified in the biological process group “brown fat cell differentiation” after a gene ontology analysis of RNA-sequencing data using DAVID software (DAVID Bioinformatics Resources). The ratio refers to the comparison of RNA-sequencing data between the PAT of P2Y_4_ KO versus WT mice, 24h after LAD ligation.

### PD-L1 is a marker of beige adipocytes up-regulated in the absence of P2Y_4_ receptor

**Figure 2:**
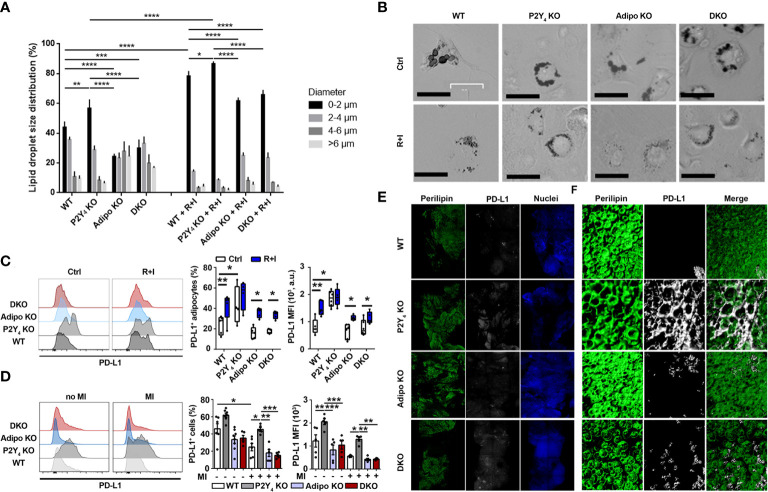
Identification of PD-L1 as a marker of beige adipocytes, upregulated in P2Y_4_ KO PAT. **(A)** Quantification of lipid droplet size in adipocytes generated from WT, P2Y_4_ KO, Adipo KO and DKO cADSCs in the presence or absence of R+I treatment. Quantification was performed using ImageJ software (n = 4). **(B)** Oil red O staining of cardiac adipose-derived stem cells (cADSC) cultures after adipogenic differentiation and after treatment with two adipocyte browning agents, rosiglitazone (1 µM) plus isoproterenol (100 µM) (R+I) (200x magnification). Adipogenic differentiation leads to big lipid droplet appearance in control (Ctrl) cADSC cultures. The additional presence of browning agents (R+I) in the differentiation medium leads to small lipid droplet appearance in cADSC cultures. **(C)** Higher PD-L1 expression in P2Y_4_ KO differentiated cADSCs. Density plots, percentage of PD-L1 positive cells and mean fluorescence intensities (MFI) obtained by flow cytometry quantification of PD-L1 expression in differentiated adipocytes isolated from PAT of WT, P2Y_4_ KO, Adipo KO and DKO mice, with or without R+I (n = 4-6). **(D)** Flow cytometry analysis showing increased level of PD-L1 expression in P2Y_4_ KO PAT. Density plots, percentage of PD-L1 positive cells and mean fluorescence intensities (MFI) detected by flow cytometry in PAT of sham (no MI) or ischemic (MI) WT, P2Y_4_ KO, Adipo KO and DKO mice, 24h post-MI (n = 5-7). **(E, F**). Loss of P2Y_4_ leads to increased PD-L1 immunohistological staining in ischemic PAT. **(E)** Immunofluorescent stainings of perilipin lipid droplet marker, and PD-L1 performed on optically cleared whole-mounted PAT of WT, P2Y_4_ KO, Adipo KO and DKO ischemic mice, 24h post-MI (5X magnification). **(F)** Representative confocal microscopy reconstructions of stained and optically cleared whole-mounted PAT (20X magnification). Data represent mean ± SEM. *p < 0.05; **p < 0.01; ***p < 0.001; ****p < 0.0001.

PD-L1 is a well-known anti-inflammatory protein, also reported as an activation-independent marker of brown adipocytes (16), which are characterized by small lipid droplets. Cardiac adipose-derived stem cells (cADSCs) were isolated from PAT of WT, P2Y_4_ KO, adiponectin KO and P2Y_4_/adiponectin double KO mice. After 3 days in proliferation medium, cADSCs were submitted to adipogenic differentiation for 7 days in a medium containing 50 μM indomethacin, 1 μM dexamethasone, 0.5 mM isobutylmethylxanthine, and 5 μg/ml insulin, and in the presence or the absence of two browning agents, rosiglitazone (1 µM) and isoproterenol (100 µM) (R+I). Oil red O staining of ADSC cultures showed a classical adipogenic differentiation with the presence of big lipid droplets in control ADSC cultures (Ctrl) of WT, P2Y_4_ KO, adiponectin KO (Adipo KO) and P2Y_4_/adiponectin double KO (DKO) mice (**Figure 2A**). In the presence of R+I, small lipid droplets were observed in the differentiated ADSC cultures, especially in WT and P2Y_4_ KO ADSCs, confirming the browning action of the two agents (**Figures 2A, B**). Lipid droplet size quantification showed that adipocytes differentiated from P2Y_4_ KO cADSCs had more small lipid droplets (0-2 μm), compared to adipocytes generated from WT cADSCs (**Figure 2A**). On the contrary, we observed less small lipid droplets (0-2 μm) in adiponectin KO and DKO cADSCs, compared to adipocytes generated from WT cADSCs (**Figure 2A**). Treatment with rosiglitozone + isoproterenol (R+I) increased the percentage of the smallest lipid droplets (0-2 μm) in all differentiated ADSC cultures, with adipocytes generated from P2Y_4_ KO cADSCs having the highest proportion of small lipid droplets (**Figure 2A**). Furthermore, R+I treatment was used to analyze PD-L1 expression after adipocyte browning (**Figure 2C**). We demonstrated by flow cytometry that PD-L1 expression was higher on adipocytes generated from P2Y_4_ KO ADSCs than from WT ADSCs (**Figure 2C**). Interestingly, small lipid droplets were already observed in P2Y_4_ KO cultures without R+I treatment (**Figures 2A, B**), corresponding to a higher PD-L1 level in P2Y_4_ KO than in WT cultures (**Figure 2C**).

In infarcted mice, flow cytometry experiments showed that PD-L1 level was down-regulated 24h after MI in WT mice, but expression level of PD-L1 was significantly higher in PAT of P2Y_4_ KO mice, than in PAT of WT, adiponectin KO and DKO PAT (**Figure 2D**). Immunohistological stainings of perilipin, a lipid droplet marker, and PD-L1 were then performed on whole-mounted PAT of WT, P2Y_4_ KO, adiponectin KO and DKO ischemic mice, 24h post-MI (**Figure 2E**). Confocal microscopy reconstructions of perilipin and PD-L1 stainings were also made at higher magnification on optically cleared whole mounted PAT of ischemic mice, 24h post-MI (**Figure 2F**). PD-L1 expression was much higher in P2Y_4_ KO PAT than in PAT of WT, adiponectin KO or DKO PAT and perilipin/PD-L1 co-staining regions were observed in the ischemic PAT (**Figure 2E, F**).

### Loss of P2Y_4_ receptor induces macrophage polarization in ischemic PAT

**Figure 3:**
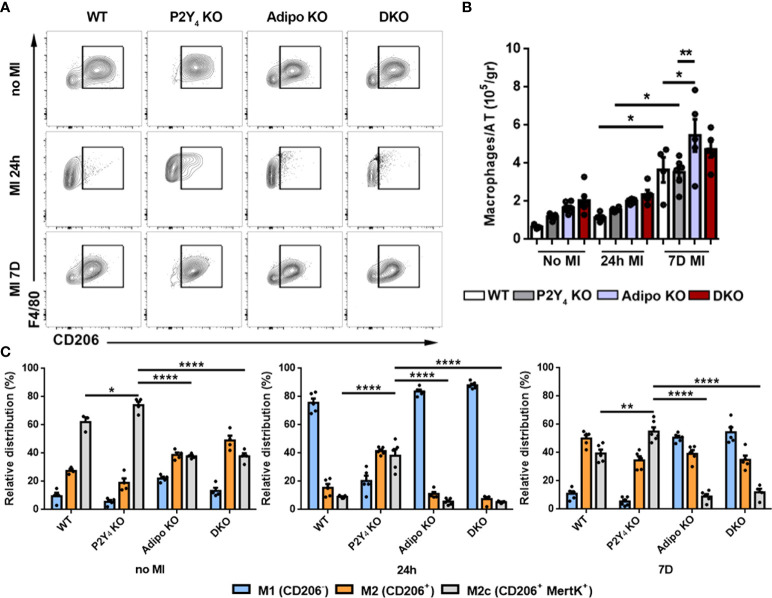
Loss of P2Y_4_ induces anti-inflammatory macrophage polarization in ischemic PAT. **(A)** Flow cytometry data are shown as representative dotplots for F4/80 and CD206, a marker of M2 macrophages, quantified in PAT of WT, P2Y_4_ KO, adiponectin KO (Adipo KO) and P2Y_4_/adiponectin double KO (DKO) mice, without myocardial infarction (no MI), or 24h or 7 days (7D) after LAD ligation. **(B)** Histograms representing increase of F4/80^+^ macrophages after LAD ligation in PAT of WT, P2Y_4_ KO, Adipo KO and DKO mice (normalized to adipose tissue (AT) weight) (n = 4-5). **(C)** Flow cytometry quantification of M1, M2 and M2c macrophage populations in PAT using CD206 and MertK, a marker of M2c anti-inflammatory macrophages. Data are expressed as relative distribution of CD206^-^, CD206^+^, and CD206^+^ MertK^+^ macrophage populations within the PAT, before and 24h or 7D after MI (n = 4-6). Data represent mean ± SEM. *p < 0.05; **p < 0.01; ****p < 0.0001.

We examined the inflammatory response in WT, P2Y_4_ KO, adiponectin KO and DKO mice subjected to LAD ligation. PAT was collected from these ischemic mice to perform flow cytometry experiments, 24h or 7 days after LAD ligation (**Figures 3A-C**). As expected, we observed increased numbers of macrophages (CD45^+^, CD11b^+^, F4/80^+^, Ly6G^-^ and CD19^-^) in PAT in adiponectin KO mice than in WT and P2Y_4_ KO ischemic mice, 7 days post-MI (**Figure 3B**). Overexpression of adiponectin and factors released by beige adipocytes might promote a polarization of macrophages with anti-inflammatory properties in P2Y_4_ KO mice. To investigate this hypothesis, we analyzed macrophage polarization in PAT of mice without MI, or 24h and 7 days post-MI (**Figure 3C**). We observed that M2 macrophages, expressing mannose receptor CD206, a macrophage activation marker, were mainly detected in PAT of mice without MI or 7 days post-MI, whereas M1 (CD206 negative) macrophages were the major population detected after 24h of MI, except in P2Y_4_ KO PAT (**Figure 3C**). Effectively, the negative effect of MI on CD206^+^ macrophage level observed in WT, adiponectin KO and DKO mice, 24 h post-MI, was not observed in P2Y_4_ KO mice (**Figure 3C**). The anti-inflammatory M2c macrophage population characterized by MertK (myeloid-epithelial-reproductive tyrosine kinase) and CD206 expression was also quantified in the PAT of WT, P2Y_4_ KO, adiponectin KO and DKO mice (**Figure 3C**). Interestingly, induction of MI by LAD ligation decreased M2c population in all tested mice but a higher percentage of M2c macrophages was observed in P2Y_4_ KO PAT, compared with WT, adiponectin KO mice and DKO mice, 24h and 7 days post-MI (**Figure 3C**).

### P2Y_4_ receptor regulates post-MI lymphoid cluster expansion and T cell polarization in PAT

**Figure 4:**
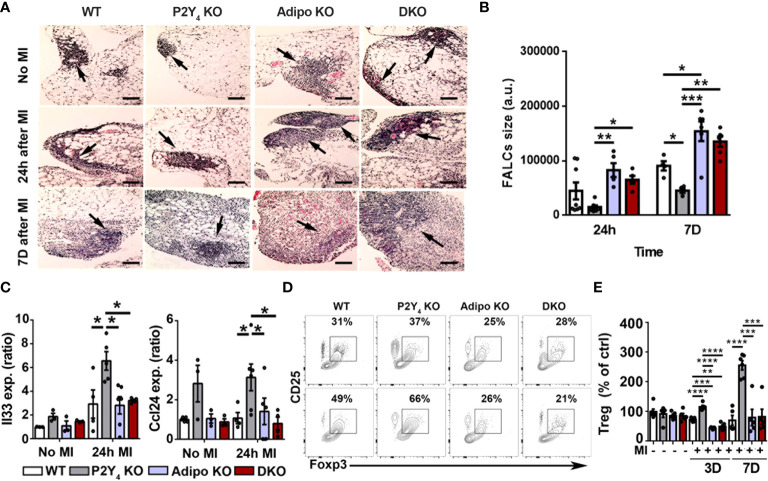
Lymphoid cluster reduced expansion and T cell polarization in ischemic PAT of P2Y_4_ mice. **(A)** Representative pictures of hematoxylin/eosin staining showing FALCs (lymphoid clusters; some are indicated with black arrows) observed in PAT of WT, P2Y_4_ KO, Adipo KO and DKO mice, with or without MI (200x magnification) (scale bar = 50μm). **(B)** Quantification of mean FALCs size detected in PAT 24 hours (24h) or 7 days (7D) after MI (a.u., arbitrary units) (n = 5). **(C)** mRNA level quantification of Il33 and Ccl24 expression in PAT of WT, P2Y_4_ KO, Adipo KO and DKO mice, without MI or 24h post-MI (relative expression normalized to Rpl32 housekeeping gene)(n = 3-6). **(D)** CD25-Foxp3 dot plots representing the gating used for flow cytometry quantification of regulatory lymphocyte population in PAT of mice without MI (upper dot plots) and 7 days post-MI (lower dot plots). **(E)** Percentage of regulatory lymphocytes in WT, P2Y_4_ KO, Adipo KO and DKO PAT from mice without MI, and 3 days (3D) or 7 days (7D) after MI (normalized to Treg level in control (ctrl) WT PAT) (n = 5-6). Data represent mean ± SEM. *p < 0.05; **p < 0.01; ***p < 0.001; ****p < 0.0001.

To investigate further post-MI leukocyte regulation, we investigated FALCs expansion in P2Y_4_ KO PAT. Hematoxylin-eosin staining of ischemic PAT sections was done to analyze lymphoid clusters and quantify FALCs size in WT, P2Y_4_ KO, adiponectin KO and DKO ischemic mice. FALCs are known to expand in the ischemic PAT after LAD ligation, reflecting the post-ischemic inflammatory response. A reduced amount of large FALCs was detected in P2Y_4_ KO PAT, even before LAD ligation, compared to WT PAT (**Figure 4A**). In contrary, a higher number of large FALCs was observed in adiponectin KO and DKO PAT (**Figure 4A**). Mean FALCs size was quantified in PAT sections of ischemic mice, 24 h and 7 days post-MI (**Figure 4B**). Smaller FALCs were observed in PAT of P2Y_4_ KO mice, compared with PAT of WT mice, and very large FALCs are observed in the absence of adiponectin (**Figures 4A, B**).

We also observed that genes associated with the presence of beige adipocytes and with recruitment and maintenance of regulatory lymphocytes in adipose tissues, such as Il33 and Ccl24, were upregulated in PAT of P2Y_4_ KO ischemic mice compared with WT, adiponectin KO and DKO mice(**Figure 4C**). The overexpression of Il33 and Ccl24 in PAT of P2Y_4_ KO mice is observed after 24h after LAD ligation (**Figure 4C**). To strengthen our findings, we investigated whether macrophage polarization and FALCs size data were correlated with a change in T regulatory (Treg) leukocyte population (**Figures 4D, E**). **Figure 4D** shows CD25-Foxp3 flow cytometry dot plots used for the gating of regulatory lymphocyte population in PAT of mice without MI (upper dot plots) and 7 days post-MI (lower dot plots). Flow cytometry data showed an increased number of regulatory T cells (CD3^+^ CD25^+^ Foxp3^+^) in P2Y_4_ KO PAT, compared with WT, adiponectin KO and DKO PAT, 3 and 7 days post-MI (**Figure 4E**).

### Increased T cell apoptosis observed in PAT of P2Y_4_ KO ischemic mice

**Figure 5:**
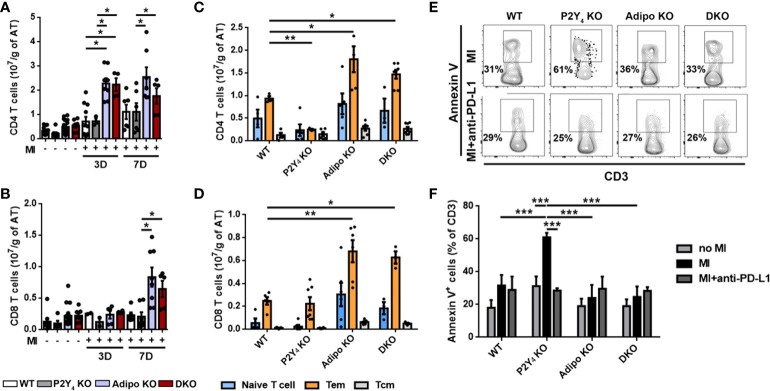
T cell apoptosis is increased in ischemic PAT of P2Y_4_ KO mice. **(A, B)** Flow cytometry quantification of CD4 and CD8 T lymphocytes in PAT of WT, P2Y_4_ KO, Adipo KO and DKO mice, 3 or 7 days (3D or 7D) post-MI, normalized to adipose tissue (AT) weight (g)). **(C, D)** Quantification of naive T cells, effector memory T cells (Tem) and central memory T cells (Tcm) by quantification of CD44 and CD62L in ischemic PAT, 7 days post-MI (n = 5-9). **(E)** Representative flow cytometry dotplots for annexin V^+^ CD3^+^ lymphocytes in PAT WT, P2Y_4_ KO, Adipo KO and DKO mice, 7 days post-MI. Mice were injected intraperitoneally or not, with anti-PD-L1 blocking antibody (10 µg/g) (PD-L1), 1h before LAD ligation then at days 1, 3 and 5 post-LAD ligation. **(F)** Flow cytometry quantification of annexin V^+^ apoptotic T cells in PAT of WT, P2Y_4_ KO, Adipo KO and DKO mice (percentage compared to total CD3 positive cells), 7 days post-MI mice or in sham mice (no MI). Ischemic mice were injected intraperitoneally or not, with anti-PD-L1 blocking antibody (10 µg/g), 1h before LAD ligation, then at days 1, 3 and 5 post-LAD ligation (n = 5-6). Data represent mean ± SEM. *p < 0.05; **p < 0.01; ***p < 0.001.

To further investigate the importance of PD-L1 in our model, we studied the effect of P2Y_4_ and adiponectin loss, on T cell expansion in PAT, after MI. CD4^+^ and CD8^+^ T cell numbers were comparable in PAT from WT and P2Y_4_ KO mice, 3 and 7 days after MI (**Figures 5A, B**). On the contrary, CD4^+^ and CD8^+^ T cell numbers in PAT were significantly higher in adiponectin KO and double KO ischemic mice than in WT ischemic mice (**Figures 5A, B**). More particularly, CD4^+^ T cell number was increased 3 and 7 days after LAD ligation in PAT from adiponectin KO and double KO ischemic mice, compared with PAT from WT ischemic mice (**Figure 5A**). CD8^+^ T cell number was increased only 7 days after LAD ligation in PAT from adiponectin KO and double KO ischemic mice, compared with PAT from WT ischemic mice (**Figure 5B**).

We analyzed CD4^+^ and CD8^+^ T cells freshly isolated from PAT, for CD44 and CD62L (L-selectin) co-expression (**Figures 5C, D**). Evaluation of CD44 and CD62L co-expression level is considered as a standard procedure to categorize T cells into memory and naïve phenotypes (17). CD44 is a prominent activation marker distinguishing memory and effector T cells from their naïve counterparts. The CD44^low^ CD62L^+^ population is considered as naïve T cells, the CD44^high^ CD62L^+^ population considered as central memory T cells (Tcm), and the CD44^high^ CD62L^-^ population considered as effector memory T cells (Tem) (17). Naïve T cells, Tem and Tcm were normalized to PAT mass and are shown in **Figures 5C, D**. A significant decrease in CD4^+^ Tem population was significantly observed in PAT of P2Y_4_ KO mice, compared with that observed in PAT of WT mice (**Figure 5C**). On the contrary, a significant increase in CD4^+^ and CD8^+^ Tem level was observed in PAT from adiponectin KO and P2Y_4_/adiponectin double KO ischemic mice, compared with that observed in PAT from WT mice (**Figures 5C, D**). The difference observed in naïve T cell and Tcm populations in WT, P2Y_4_ KO, adiponectin KO and double KO mice were not statistically significant (**Figures 5C, D**).

Interestingly, increased level of Tem lymphocytes has been associated with patients suffering from CAD such as atherosclerosis and myocardial infarction (18). We decided to investigate whether the limited expansion of Tem lymphocytes in PAT of P2Y_4_ KO mice is the result of a proliferation defect or apoptosis, after MI. Ki-67 analysis in T cell populations revealed no significant differences between their proliferation in P2Y_4_ KO and WT PAT (data not shown). To investigate T cell apoptosis, PAT was harvested from ischemic mice after 7 days of MI, and used for flow cytometry analysis using annexin V and CD3 antibodies (**Figures 5E, F**). We observed a significant increase of annexin V^+^ apoptotic T cells in P2Y_4_ KO PAT, compared to WT PAT, whereas apoptotic T cell percentage remained unchanged in PAT of adiponectin KO and DKO mice (**Figures 5E, F**).

To investigate the potential involvement of PD-L1 in T lymphocyte regulation in ischemic PAT after MI, the effect of PD-L1 inhibition was evaluated on the apoptosis of T cells. Mice were injected intraperitoneally with an anti-PD-L1 blocking antibody (10 µg/g), 1h before LAD ligation and at days 1, 3 and 5 post-MI during MI onset. Flow cytometry experiments revealed that PD-L1 blocking antibody abolished the increase in apoptotic T cells observed in PAT of P2Y_4_ KO mice, 7 days post-MI (**Figures 5E, F**).

### Role of PD-L1 in reduced cardiac inflammation and fibrosis observed in P2Y_4_ KO ischemic hearts

**Figure 6:**
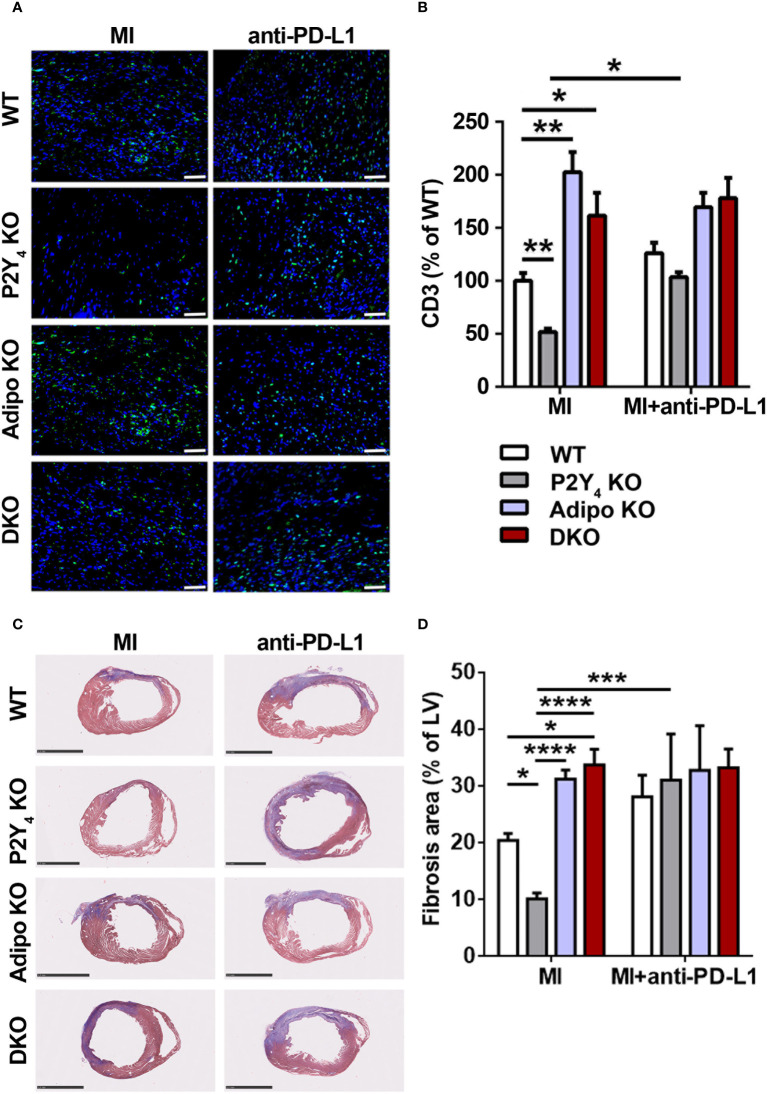
Anti-PD-L1 blocking antibody inhibits the reduction of T cell infiltration and fibrosis observed in ischemic P2Y_4_ KO hearts. **(A)** Representative T cell staining (CD3) in myocardial infarct sections of WT, P2Y_4_ KO, adiponectin KO (Adipo KO) or P2Y_4_/adiponectin double KO (DKO) mice, 7 days after LAD ligation using anti-CD3 (green) and Hoechst in blue (20x magnification) (scale bar = 50μm). Mice were intraperitoneally injected or not (MI), with anti-PD-L1 blocking antibody (10 µg/g), 1h before LAD ligation then at days 1, 3 and 5 post-LAD ligation. **(B)** CD3^+^ cell quantification in representative counting surface/field (0.1 mm^2^) of myocardial infarct sections, 7 days after LAD ligation. Data are expressed as percentages compared to T cell number in WT ischemic mice and obtained using ImageJ software by examining 30 fields per heart at 100x magnification (n = 5-6). **(C)** Representative Masson’s trichrome staining of cardiac fibrosis (in blue) in WT, P2Y_4_ KO, Adipo KO or DKO mice, 7 days after LAD ligation, injected or not with anti-PDL1 blocking antibody (10 µg/g)) (scale bar = 2.5 mm). **(D)** Fibrosis area represents collagen staining in blue quantified by color image analyser ImageJ in left ventricle (LV) and expressed as percentages of total LV surface (2.5x magnification) (n = 5-6). Data represent mean ± SEM. *p < 0.05; **p < 0.01; ***p < 0.001; ****p < 0.0001.

The presence of beige adipocytes and regulatory leukocyte populations, as well as reduced FALCs expansion in PAT of P2Y_4_ KO mice, could greatly contribute to protection against myocardial infarction observed in these mice. We decided to analyze T lymphocyte infiltration in the ischemic heart of WT, P2Y_4_ KO, adiponectin KO and P2Y_4_/adiponectin double KO mice, after PD-L1 blockade. The number of cardiac CD3^+^ lymphocytes quantified 7 days after MI, was significantly lower in P2Y_4_ KO hearts than in WT hearts (**Figures 6A, B**). Interestingly, T lymphocyte reduction in P2Y_4_ KO ischemic hearts was no more observed after PD-L1 inhibition by intraperitoneal injection of anti-PD-L1 blocking antibody (10 µg/g), 1h before LAD ligation, and then at days 1, 3 and 5 post-LAD ligation (**Figures 6A, B**).

We also analyzed the effect of anti-PD-L1 blocking antibody injection on cardiac fibrosis by Masson’s trichrome staining of infarcted hearts (**Figures 6C, D**). The reduction in fibrosis area observed in P2Y_4_ KO ischemic hearts, compared with WT ischemic hearts, was no more observed in ischemic hearts of anti-PD-L1-injected P2Y_4_ KO mice (**Figure 6D**). The increase in cardiac fibrosis observed in adiponectin KO and DKO hearts compared with WT hearts, was no more observed in anti-PD-L1-injected adiponectin KO and DKO hearts mice (**Figure 6D**).

## Discussion

The pericardial adipose tissue contains a high density of FALCs that might play a relevant role in regulating cardiac response to ischemia. After myocardial infarction, leukocytes, organized in FALCs in PAT, are generally considered detrimental. They are activated a few hours after onset of MI and contribute to acute tissue injury by promoting neutrophils mobilization from the bone marrow (4). The communication between FALCs and adipocytes and their role in infarct healing has been largely neglected so far.

The quantity of adipose tissue exceeds the normal value during obesity and is considered as a negative marker during cardiovascular events. Although loss of P2Y_4_ was associated previously with an increase in PAT mass, smaller infarcts are observed in P2Y_4_ KO mice, as well as adiponectin overexpression leading to endothelin-1 decrease (14, 15). MI induced expansion of FALCs in PAT of WT ischemic mice (4). Interestingly, we showed here that FALCs expansion is amplified in adiponectin KO ischemic PAT and inhibited in P2Y_4_ KO ischemic PAT, in which mean FALCs size stays at a comparable level to control mice without infarction. We have previously demonstrated that mouse P2Y_4_ receptor, expressed in cADSC and cardiac adipocytes, is a negative regulator of cADSC adipogenic differentiation (15). Our data showed here an increase in beige adipocytes within the PAT of P2Y_4_ KO ischemic mice. Previous studies described the cardioprotective role of beige adipose tissue and reported its effect on metabolism improvement (8, 9). Modulation of cardiac and vascular adipose tissue to increase the proportion of beige adipocytes could be a feasible way to improve local inflammation and reduce cardiovascular risk. However, whereas there are methods to ‘brown’ fat in rodents, few of these can be translated to the human population.

Thanks to the use of P2Y_4_ KO and adiponectin KO mice, we investigated possible interactions between adipocytes and FALCs to regulate post-MI inflammatory response. Among positive markers of beige adipocytes such as UCP1, their expression of PD-L1 was not yet demonstrated. However, PD-L1 was reported as a marker of brown adipose tissue (16). We identified PD-L1 expression in cultures of cardiac adipocytes obtained after cADSC adipogenic differentiation. Their stimulation with rosiglitazone plus isoproterenol is known to induce browning of adipocytes, characterized by small lipid droplets, and increased their expression of PD-L1. *In vitro* data showing higher PD-L1 expression in P2Y_4_ KO than in WT adipocyte cultures were correlated with more pronounced *in vivo* PD-L1 expression in P2Y_4_ KO ischemic PAT than in WT ischemic PAT.

After an acute inflammatory phase mainly driven by neutrophils and pro-inflammatory M1 macrophages, these leukocytes are replaced by reparative M2 macrophages, which facilitate wound healing and regeneration by promoting myofibroblast accumulation, collagen deposition, and angiogenesis. A proper inflammatory response involves accurate clearance of dead cells, which is a prerequisite for favorable MI healing. In post-MI repair, macrophages expressing M2c marker MertK play a crucial role in the clearance of cell debris (1). The present study reveals that anti-inflammatory M2c macrophage polarization is induced in the PAT of P2Y_4_ KO ischemic mice.

The identification of macrophage polarization in P2Y_4_ KO PAT from a pro-inflammatory M1 phenotype to an anti-inflammatory M2 phenotype is very promising. M2c macrophages are involved in various pro-regenerative functions. Interestingly, increased presence of another anti-inflammatory leukocyte population, Treg, was also observed in ischemic P2Y_4_ KO PAT. Adipose tissue has already been described as a reservoir of Foxp3^+^ regulatory T cells (19). These cells are abundant in the visceral adipose tissue of normal diet mice, but their proportion is greatly reduced in insulin-resistant animal models of obesity (19). The depletion of Treg cells leads to a pro-inflammatory visceral adipose tissue state and the worsening of metabolic control in obese mice (19). Matarese et al. explained the dramatic reduction in Treg cells in visceral adipose tissue of obese individuals by the inhibitory effect of leptin on Treg cell proliferation (20). Supernatants of M2c macrophages can robustly induce Foxp3 expression in human CD4 T cells having immunosuppressive activity (21). Our data support that adipocytes are crucial regulators of the microenvironment driving leukocyte polarization and regulating adipose tissue inflammatory state.

Adiponectin can prime human monocyte differentiation into anti-inflammatory M2 macrophages (22). In addition, it has been demonstrated that adiponectin-treated antigen-presenting cells can promote Treg cell expansion *via* the PD-1/PD-L1 pathway (23, 24). Effectively, adiponectin can reduce costimulatory molecules and MHC II, and increase PD-L1 expression on dendritic cells, which impairs their stimulatory capacity (23). AdipoR1 activation by adiponectin in murine Tregs can also mediate the release of IL-10 (25), which is required for PD-L1 expression on Treg (26).

Tumor cells evade immune surveillance by upregulating the surface expression of PD-L1, which interacts with programmed death-1 (PD-1) receptor on T cells to elicit the immune checkpoint response. Our data support that increased expression of PD-L1 in fat depots, preferentially beige adipose tissue, might contribute to the accumulation of Treg cells observed in P2Y_4_ KO ischemic PAT. The accumulation of Treg cells in adipose tissue is dependent on a combination of cytokine expression in the microenvironment (27). Relevant cytokines involved in Treg activation and recruitment are elevated in P2Y_4_ KO mice after MI, such as IL33 that stimulates Treg cells and regulates T cell expansion to reduce the formation of atherosclerosis plaque (28). In cardiac xenotransplantation, IL33 prolongs transplant survival time by increasing the number of myeloid-derived suppressor and Treg cells (29). Interestingly, our data showed that the levels of M2c, Treg cells and IL33 are higher in the PAT of P2Y_4_ KO ischemic mice than in the PAT of WT ischemic mice. The cardioprotective role of Treg cells in heart disease was demonstrated using T cell adoptive transfer experiments to protect the heart against MI in mice (30). Although a certain amount of T cells is required for cardiac healing and priming of myofibroblasts in the infarct area, excessive cardiac T cell influx may promote an unfavorable remodeling process (31).

We investigated further the involvement of PD-L1 in the cardioprotection and reduced cardiac inflammation observed in P2Y_4_ KO mice. Overexpression of PD-L1 in P2Y_4_ KO PAT could explain reduced post-ischemic FALCs expansion and lower leukocyte recruitment to the infarcted heart. P2Y_4_ KO and adiponectin KO mice were intraperitoneally injected with a blocking antibody against PD-L1 during MI onset to perform an analysis of ischemic hearts after 7 days. Flow cytometry experiments revealed that anti-PD-L1 neutralizing antibody totally inhibited the increase of apoptotic T cells observed in FALCs of ischemic P2Y_4_ KO PAT. We also demonstrated a lower number of effector memory T lymphocytes (Tem) in P2Y_4_ KO FALCs. Tem lymphocyte increase has been identified in patients suffering from CAD such as atherosclerosis and myocardial infarction (18). Interestingly we observed that the intraperitoneal injection of anti-PD-L1 blocking antibody was sufficient to abolish the reduction of T cell infiltration observed in P2Y_4_ KO infarcted hearts, 7 days post-MI. Moreover, the reduction in fibrosis area observed in P2Y_4_ KO ischemic hearts, compared with WT ischemic hearts, was no more observed in ischemic hearts of anti-PD-L1-injected P2Y_4_ KO mice, supporting the injection can abolish the cardioprotective effect linked to P2Y_4_ loss. We conclude that overexpression of PD-L1 and adiponectin in P2Y_4_ KO ischemic mice is able to reduce post-ischemic cardiac inflammation.

Our novel findings showing that reduced inflammation was lost in P2Y_4_ KO mice after PD-L1 inhibition during MI onset highlight a determinant role of PD-L1 in the modulation of post-MI outcome and thus protection against myocardial infarction in these mice. The data obtained by parallel use of P2Y_4_ KO, adiponectin KO and P2Y_4_/adiponectin double KO mice in the present study support the importance of adiponectin overexpression in the anti-inflammatory effects resulting from P2Y_4_ loss. Reduced FALCs expansion in PAT, as well as lower cardiac inflammation, observed in P2Y_4_ KO ischemic mice, were not observed in P2Y_4_/adiponectin double KO ischemic mice.

The present study reinforces the notion that adipocytes have previously escaped recognition as important inflammatory regulators, and illustrate the communication between FALCs and PAT. We previously pointed out the importance of this communication to modulate granulopoiesis, and outcome after MI (4). The regulation of adiponectin and PD-L1 operates during cardiac inflammation and can integrate crucial effector mechanisms, limiting myocardial infarction and its complications.

In conclusion, a better knowledge of complex cardiac immune regulations under homeostatic and pathophysiological conditions might further enhance our understanding of cardiac stress responses to severe events such as an acute MI. There are pathophysiological arguments and clinical findings in the cardiovascular field supporting the inclusion of anti-inflammatory medications in the therapeutic arsenal against myocardial infarction. The present study could lead to new approaches for more targeted and better balanced anti-inflammatory therapies to improve MI outcome.

## Data availability statement

The RNA-seq data presented in this study are deposited in the GEO repository, accession number GSE211768, https://www.ncbi.nlm.nih.gov/geo/query/acc.cgi?acc=GSE211768.

## Ethics statement

The animal study was reviewed and approved by CEBEA (Commission d’éthique du bien-être animal), Free University of Brussels (current approved protocols 659N and 714N).

## Author contributions

MH and DC designed research study and analyzed data. MH, EDV, MB, and LDR conducted experiments and acquired data. MH and DC wrote the manuscript. All authors contributed to the article and approved the submitted version.

## Funding

This work was supported by Research Project and Research Credit of the Fonds National de la Recherche Scientifique of Belgium (J.0060.18 CDR grant), by an ATIMI (Attract Brains for Brussels, Belgium) grant of Innoviris Brussels (2019-BFB-106 ATIMI grants), by the Fonds pour la Chirurgie Cardiaque, by the Fund Lokumo, King Baudouin Foundation, Belgium (2017-B7131100-207336 grant), by the Deutsche Forschungsgemeinschaft grants SFB1123/Z1 and INST409/150-1FUGG and by the Fonds et Crédit d’Encouragement à la Recherche (F.E.R./C.E.R., Free University of Brussels (U.L.B.)). MH was supported by ATIMI (Attract Brains for Brussels, Belgium) grants of Innoviris Brussels. EDV was supported by the F.R.I.A., Fonds National de la Recherche Scientifique, Belgium. LDR is supported by U.L.B., Belgium. DC is Senior Research Associate of the Fonds National de la Recherche Scientifique (F.N.R.S.). The funders had no role in study design, data collection and analysis, decision to publish, or preparation of the manuscript.

## Acknowledgments

The authors thank Frédérick Libert for technical advice and help related to RNA-sequencing experiments.

## Conflict of interest

The authors declare that the research was conducted in the absence of any commercial or financial relationships that could be construed as a potential conflict of interest.

## Publisher’s note

All claims expressed in this article are solely those of the authors and do not necessarily represent those of their affiliated organizations, or those of the publisher, the editors and the reviewers. Any product that may be evaluated in this article, or claim that may be made by its manufacturer, is not guaranteed or endorsed by the publisher.
