# Corrigendum: Central role of PD-L1 in cardioprotection resulting from P2Y_4_ nucleotide receptor loss

**DOI:** 10.3389/fimmu.2022.1061958

**Published:** 2022-10-28

**Authors:** Michael Horckmans, Esteban Diaz Villamil, Mariaelvy Bianchini, Lucas De Roeck, Didier Communi

**Affiliations:** ^1^ Institute of Interdisciplinary Research, Institut de Recherche Interdisciplinaire en Biologie Humaine et Moléculaire (IRIBHM), Free University of Brussels, Brussels, Belgium; ^2^ Institute for Cardio-vascular Prevention, Ludwig-Maximilians-Universität (LMU), Munich, Germany

**Keywords:** cardioprotection, cardiac adipose tissue, PD-L1, P2Y receptor, adiponectin, ischemia

In the published article, there was an error in the author list, and author Mariaelvy Bianchini was erroneously excluded. The corrected author list appears below.

Michael Horckmans^1^, Esteban Diaz Villamil^1^, Mariaelvy Bianchini^2^, Lucas De Roeck^1^, Didier Communi^1^

This author’s affiliation has been added: “Institute for Cardio-vascular Prevention, Ludwig-Maximilians-Universität (LMU), Munich, Germany”.

Accordingly, a correction has been made to **Author contributions**.

This sentence previously stated:

“MH and DC designed research study and analyzed data; MH, EDV, and LDR conducted experiments and acquired data, M.H. and D.C. wrote the manuscript.”

The corrected sentence appears below:

“MH and DC designed research study and analyzed data; MH, EDV, MB, and LDR conducted experiments and acquired data, MH and DC wrote the manuscript.”

A correction has also been made to **Acknowledgments**.

This sentence previously stated:

“The authors thank Mariaelvy Bianchini and Frédérick Libert for technical advice and help related to respectively, PD-L1 immunofluorescent reconstructions and RNA-sequencing experiments.”

The corrected sentence appears below:

“The authors thank Frédérick Libert for technical advice and help related to RNA-sequencing experiments.”

In the published article, there was also an error in the legend for **Figure 2, panels E–F** as published, due to errors in microscopy device and magnification.

**Figure 2:**
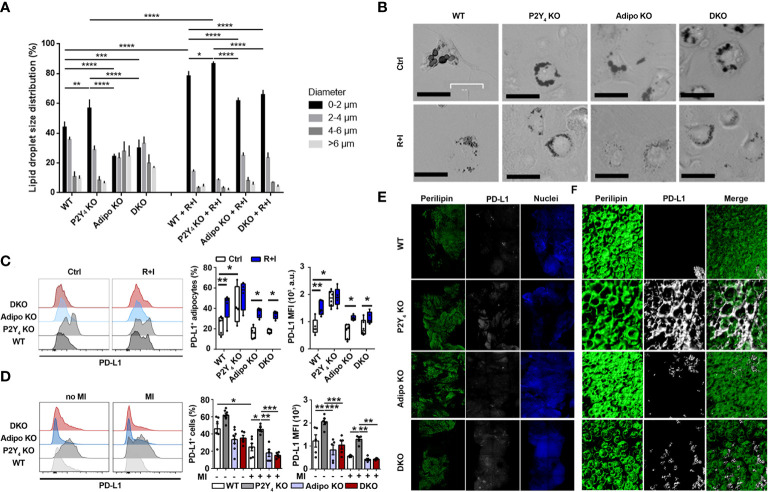
Identification of PD-L1 as a marker of beige adipocytes, upregulated in P2Y_4_ KO PAT. **(A)** Quantification of lipid droplet size in adipocytes generated from WT, P2Y_4_ KO, Adipo KO and DKO cADSCs in the presence or absence of R+I treatment. Quantification was performed using ImageJ software (n = 4). **(B)** Oil red O staining of cardiac adipose-derived stem cells (cADSC) cultures after adipogenic differentiation and after treatment with two adipocyte browning agents, rosiglitazone (1 µM) plus isoproterenol (100 µM) (R+I) (200x magnification). Adipogenic differentiation leads to big lipid droplet appearance in control (Ctrl) cADSC cultures. The additional presence of browning agents (R+I) in the differentiation medium leads to small lipid droplet appearance in cADSC cultures. **(C)** Higher PD-L1 expression in P2Y_4_ KO differentiated cADSCs. Density plots, percentage of PD-L1 positive cells and mean fluorescence intensities (MFI) obtained by flow cytometry quantification of PD-L1 expression in differentiated adipocytes isolated from PAT of WT, P2Y_4_ KO, Adipo KO and DKO mice, with or without R+I (n = 4-6). **(D)** Flow cytometry analysis showing increased level of PD-L1 expression in P2Y_4_ KO PAT. Density plots, percentage of PD-L1 positive cells and mean fluorescence intensities (MFI) detected by flow cytometry in PAT of sham (no MI) or ischemic (MI) WT, P2Y_4_ KO, Adipo KO and DKO mice, 24h post-MI (n = 5-7). **(E, F)**. Loss of P2Y_4_ leads to increased PD-L1 immunohistological staining in ischemic PAT. **(E)** Immunofluorescent stainings of perilipin lipid droplet marker, and PD-L1 performed on optically cleared whole-mounted PAT of WT, P2Y_4_ KO, Adipo KO and DKO ischemic mice, 24h post-MI (5X magnification). **(F)** Representative confocal microscopy reconstructions of stained and optically cleared whole-mounted PAT (20X magnification).

The legend previously said: **“E.** Immunofluorescent stainings of perilipin lipid droplet marker, and PD-L1 performed on PAT sections of WT, P2Y_4_ KO, Adipo KO and DKO ischemic mice, 24h post-MI (10x magnification). **F.** Representative two-photon laser scanning microscopy reconstructions of stained PAT sections (100x magnification).”

The corrected legend appears below.

In the published article, there was an error in the Funding statement. A funding source was missing. The correct Funding statement appears below.

## Funding

This work was supported by Research Project and Research Credit of the Fonds National de la Recherche Scientifique of Belgium (J.0060.18 CDR grant), by an ATIMI (Attract Brains for Brussels, Belgium) grant of Innoviris Brussels (2019-BFB-106 ATIMI grants), by the Fonds pour la Chirurgie Cardiaque, by the Fund Lokumo, King Baudouin Foundation, Belgium (2017-B7131100-207336 grant), by the Deutsche Forschungsgemeinschaft grants SFB1123/Z1 and INST409/150-1FUGG and by the Fonds et Crédit d’Encouragement à la Recherche (F.E.R./C.E.R., Free University of Brussels (U.L.B.)). MH was supported by ATIMI (Attract Brains for Brussels, Belgium) grants of Innoviris Brussels. EDV was supported by the F.R.I.A., Fonds National de la Recherche Scientifique, Belgium. LDR is supported by U.L.B., Belgium. DC is Senior Research Associate of the Fonds National de la Recherche Scientifique (F.N.R.S.). The funders had no role in study design, data collection and analysis, decision to publish, or preparation of the manuscript.

In the published article, there were errors in the used microscopy methodology related to PD-L1 and perilipin staining. Appropriate changes have been made in the microscopy device and method (two-photon laser scanning microscopy was replaced by confocal microscopy reconstructions) in a paragraph of the Materials and methods.

A correction has been made to **Materials and methods**, *“Immunofluorescence experiments”*, paragraph 1. These sentences previously stated:

“Frozen sections (5 µm) of mouse PAT were stained with antibodies against Programmed death ligand 1 (PD-L1) (BioLegend, San Diego, CA, USA) and perilipin (Sigma-Aldrich). For some experiments of PD-L1 and perilipin staining, two-photon laser scanning microscopy (TPLSC) was performed on a Leica SP5II MP equipped with Ti : Sa pulsed laser (Spectra-Physics, MKS Instruments, Andover, MA, USA) tuned at 800 nm.”

The corrected sentences appear below:

“Whole-mounted mouse PAT was fixed in 2% PFA, stained overnight with antibodies against Programmed death ligand 1 (PD-L1) (BioLegend, USA) and perilipin (Abcam, UK), then optically cleared with RapiClear 1.47 (SUNJin Lab, South Korea) for 6 hours. Samples were acquired as tile-scan overviews with a Leica Thunder Imager (Leica Microsystems, Germany). For higher optical resolution imaging, 3D image stacks were acquired using a Leica SP8 3X confocal microscope (Leica Microsystems, Germany) equipped with a tunable white light laser. Image 3D reconstructions were generated with Imaris 8 (Oxford Instruments, UK).”

In the published article, there were errors related to the used microscopy methodology (two-photon laser scanning microscopy was replaced by confocal microscopy reconstructions) related to PD-L1 and perilipin staining in the Results.

A correction has been made to **Results**, *“PD-L1 is a marker of beige adipocytes up-regulated in the absence of P2Y_4_ receptor”*, paragraph 2. These sentences previously stated:

“Immunohistological stainings of perilipin, a lipid droplet marker, and PD-L1 were then performed on PAT sections of WT, P2Y_4_ KO, adiponectin KO and DKO ischemic mice, 24h post-MI (**Figure 2E**). Two-photon laser scanning microscopy (TPLSM) reconstructions of perilipin and PD-L1 stainings were also made at higher magnification on PAT sections of ischemic mice, 24h post-MI (**Figure 2F**).”

The corrected sentences appear below:

“Immunohistological stainings of perilipin, a lipid droplet marker, and PD-L1 were then performed on whole-mounted PAT of WT, P2Y_4_ KO, adiponectin KO and DKO ischemic mice, 24h post-MI (**Figure 2E**). Confocal microscopy reconstructions of perilipin and PD-L1 stainings were also made at higher magnification on optically cleared whole mounted PAT of ischemic mice, 24h post-MI (**Figure 2F**).”

The authors apologize for these errors and state that they do not change the scientific conclusions of the article in any way. The original article has been updated.

## Publisher’s note

All claims expressed in this article are solely those of the authors and do not necessarily represent those of their affiliated organizations, or those of the publisher, the editors and the reviewers. Any product that may be evaluated in this article, or claim that may be made by its manufacturer, is not guaranteed or endorsed by the publisher.

